# Prevalence of disease complications and risk factor monitoring amongst diabetes and hypertension patients attending chronic disease management programmes in a South African Township

**DOI:** 10.4102/phcfm.v13i1.2997

**Published:** 2021-09-08

**Authors:** Tiny Masupe, Jeroen De Man, Sunday Onagbiye, Thandi Puoane, Peter Delobelle

**Affiliations:** 1 Department of Family Medicine and Public Health, Faculty of Medicine, University of Botswana, Gaborone, Botswana; 2 School of Public Health, Faculty of Health Sciences, University of the Western Cape, Bellville, Cape Town, South Africa; 3 Department of Family Medicine and Population Health, Centre for General Practice, University of Antwerp, Antwerp, Belgium; 4 The Africa Unit for Transdisciplinary Health Research (AUTHeR), North-West University, Potcheftroom Campus, South Africa; 5 Chronic Disease Initiative for Africa, Department of Medicine, Faculty of Health Sciences, University of Cape Town, Cape Town, South Africa; 6 Department of Public Health, Faculty of Medicine and Pharmacy, Vrije Universiteit Brussel, Brussels, Belgium

**Keywords:** diabetes complications, hypertension, illness perception, chronic disease management programmes, risk factor monitoring, self-management support

## Abstract

**Background:**

South Africa established chronic disease management programmes (CDMPs) called ‘clubs’ to ensure quality diabetes care. However, the effectiveness of these clubs remains unclear in terms of disease risk factor monitoring and complication prevention.

**Aim:**

We assessed risk factor monitoring, prevalence and determinants of diabetes related complications amongst type-2 diabetes (T2D) and hypertension (HTN) patients attending two CDMPs.

**Setting:**

Urban Township in Cape Town, South Africa.

**Methods:**

Cross-sectional survey combined with a 10-year retrospective medical records analysis of adult T2D/HTN patients attending two CDMPs, using a structured survey questionnaire and an audit tool. Statistical Software for Social Sciences (SPSS) version 25 was used to analyse risk factor monitoring and calculate prevalence of complications. Potential determinants of complications were explored through logistic regression.

**Results:**

There were 379 patients in the survey, 372 (97.9%) had HTN whilst 159 (41.9%) had T2D and HTN; 361 medical records were reviewed. Blood pressure (87.7%) and weight (86.6%) were the best monitored risk factors. Foot care (0.0% – 3.9%) and eye screening (0.0% – 1.1%) were least monitored. Nearly 22.0% of patients reported one complication, whilst 9.2% reported ≥ 3 complications. Medically recorded complications ranged from 11.1% (1 complication) to 4.2% with ≥ 3 complications. The most common self-reported and medically recorded complications were eye problems (33%) and peripheral neuropathy (16.4%), respectively. Complication occurrence was positively associated with age and female gender and negatively associated with perceived illness control.

**Conclusions:**

Type-2 diabetes and hypertension patients experienced diabetes related complications and inadequate risk factor monitoring despite attending CDMPs. Increased self-management support is recommended to reduce complication occurrence.

## Introduction

In the 1990–2019 global burden of disease study, high fasting plasma glucose and high body mass index (BMI) were recorded as the most increased risk exposures to health, whilst hypertension (HTN) was the risk factor that showed the largest increase accounting for 10.8 million deaths.^1^ Most data on non-communicable diseases (NCD) prevalence in South Africa comes from national surveys such as the South African National Health and Nutrition Examination Survey (SANHANES-1) conducted in 2012, which revealed an age-standardised prevalence of diabetes in South Africans aged 15 and above of 10.1%.^2^ Prevalence rates were higher amongst the non-white people population as well as amongst women.^3^ Additionally, an increased mortality from diabetes was reported by the Burden of Disease Research Unit at the South African Medical Research Council^4^ probably resulting from lifestyle changes, urbanisation and increasing overweight and obesity amongst South Africans.^5^ According to World Health Organization data, approximately 27.4% of men and 26.1% of women in South Africa have HTN,^6^ although a higher prevalence of up to 60% has been reported.^7^ Studies have estimated a high prevalence of diabetes complications in South Africa including any grade of retinopathy (55.4%), proliferative and pre-proliferative retinopathy (15.6%), cataracts (7.9%), peripheral neuropathy (27.6%), absent foot pulses (8.2%), and amputations (1.4%).^8^ Diabetic retinopathy was the most common complication.^9^ Nearly 80 000 years lost to disability were attributed to diabetes and its complications in South Africa in 2009.^10^

South Africa has implemented several policies to guide self-management programmes for quality diabetes care. One such policy was the establishment of chronic disease management programmes (CDMPs) called ‘clubs’, aimed at equipping patients with the necessary knowledge and self-management skills in order to reduce disease progression and complications.^11^ In these clubs, stable patients get access to medication, receive monthly blood pressure and glucose monitoring, and health education on self-management of lifestyle disease risk factors. However, the effectiveness of these clubs remains unclear in terms of chronic disease monitoring and halting the progress to complications. The Western Cape NCD audit of 2016/2017 (folder review) which included the two facilities in this study, identified poor adherence to type-2 diabetes (T2D) and HTN management.^12^ However, this audit, did not assess prevalence of disease complications and only reviewed around 45 patient folders per facility. To address this gap, this study assessed yearly monitoring for disease risk factors and estimated the prevalence and determinants of disease complications amongst T2D and HTN patients attending CDMPs in a peri-urban township in Cape Town, between 2018 and 2019.

## Methods

### Study design

This was a cross-sectional survey combined with a 10-year retrospective patient records analysis (2009–2018), conducted in March 2018.

### Setting

The study took place in two community health centres in Khayelitsha, a predominantly black African (90.5%) township in Cape Town with a recent official population for 2019/2020 estimated at 442 721 by the Western Cape government.^13^ The patients were attendees of CDMPs where they had medication review, disease control, and risk factor monitoring and health education.

### Study population and sampling strategy

The study population consisted of 379 patients with T2D and/or HTN (T2D/HTN) selected from the daily clinic attendance register in both facilities using systematic random sampling. Eligibility criteria included the following: aged between 18 and 74 years; diagnosed with T2D/HTN for at least 6 months; living in Khayelitsha and attending the CDMP at the selected facility for at least 6 months; not experiencing acute illness; and able to give informed consent. Eligible patients were asked to participate whilst waiting for medical consultation. After receiving study information, consenting patients were enrolled.

The prevalence of diabetic retinopathy was used for sample size calculation because it was well documented by a previous study. A minimum sample size of 380 was calculated using Charan and Biswas prevalence estimation formula.^14^ Each health centre provided half of the required minimum sample size. Additionally, 361 medical records matching survey participants for the period 2009–2018 were audited. The percentage of missing data per variable varied between 0.0% and 2.3%, except for waist circumference (8.1%).

### Data collection

A structured survey questionnaire, based on the World Health Orginazation - STEPwise Approach to NCD Risk Factor Surveillance (STEPS) instrument^15^ was used to collect demographic and socio-economic data, behavioural measures (diet, physical activity, tobacco and alcohol use), medical and medication history, and details of self-reported complications (eye problems, foot pain, diabetic retinopathy, diminished peripheral pulses, peripheral neuropathy and amputation). Anthropometric measures (height, weight, waist circumference), sitting blood pressure and biochemical measures (fasting plasma glucose [FPG], haemoglobin A1c [HbA1c]) were also collected.

Self-management variables of diabetes knowledge and illness perception were also measured. Knowledge was measured using the Diabetes Knowledge Scale consisting of 13 items from the validated Diabetes Knowledge Questionnaire (DKQ-24), selected based on their contextual relevance.^16^ Correct answers were scored as 1, and incorrect or ‘Don’t know’ responses as zero, yielding a total score ranging from 0 to 13. Perceived diabetes and HTN control was measured using three items selected from the Brief Illness Perception Questionnaire (B-IPQ),^17^ which is validated across 36 countries, 26 languages, several continents and a variety of illnesses including T2D and cardiovascular diseases amongst patients aged between 8 and 80 years.^18^ Selected items included the following: ‘How much control do you feel you have over your diabetes or hypertension?’ with responses ranging from ‘Absolutely no control’ (0), ‘Limited amount of control’ (1) and ‘Good amounts of control’ (2) to ‘Complete control’ (3). The other items related to the perception of treatment and reported experience of symptoms. Scores ranged from 0 to 9. Factor loadings of this construct were tested using confirmatory factor analysis, ranging from 0.62 to 0.74, and coefficient alpha was 0.72, indicating adequate internal reliability.

To assess risk factor monitoring for disease control and clinician-recorded complications, a retrospective medical audit of patient files was conducted using an audit tool designed by combining variables from relevant international, national and local guidelines,^19,20,21^ and patient facility monitoring cards, used for recording and monitoring of chronic disease care in the Western Cape. The tool captured information on self-care, knowledge, beliefs, and health education on lifestyle risk factor management (diet, exercise, smoking, alcohol use); psychological status; biometric monitoring (annual height, weight, and waist circumference); glucose control and clinical monitoring; cardiovascular risk indicators (blood pressure, total and LDL cholesterol, aspirin use); foot care; eye care (annual screening); renal function and medication management and monitoring.

### Data analysis

Data captured in the audit tools and survey questionnaires, was transferred into Redcap software hosted at the University of the Western Cape (UWC). Two field workers entered the same participant data independently and then compared their entries, enabling data quality checks during the data collection phase. Field worker supervisor and the research team undertook additional data checks (missing data, data consistency, and deviant entries) before exporting data into a Microsoft Excel spreadsheet. After cleaning, data was analysed using Statistical Package for Social Sciences (SPSS) software version 25.

The proportion of eligible patients who received risk factor monitoring annually was calculated to assess disease monitoring. Descriptive statistics were produced and the proportion of patients with specific complications was calculated to estimate prevalence rates. Multivariable logistic regression using R software was performed to explore associations between the occurrence of complications (binary dependent variable) and socio-demographic characteristics and self-management variables (independent variables). In a first model, the socio-demographic characteristics included were age, gender, education level, marital status (married or cohabiting), employment status, and income level. In a second model, self-management variables were added, including diabetes knowledge and perceived illness control, whilst socio-demographic variables and reported intake of oral anti-diabetics were included as control variables. The occurrence of complications corresponded to ‘having any self-reported complication’ or ‘having any clinician recorded complication’.

### Ethical considerations

Approval was granted by the University Biomedical Research Ethics Committee and Western Cape Provincial health authorities (reference: WC_2017RP50_730). Health facilities participating in the study also granted permission to access the facilities, patients and their medical records.

## Results

Most patients (*n* = 372, 98.0%) had HTN, with 159 patients (42.0%) having T2D/HTN (see Table 1). Most respondents were female (*n* = 308, 81.3%) and the mean age was 55 (±10.4 years). Two fifths (*n* = 160, 42.2%) had no or primary level education, and over half were unemployed (*n* = 210, 55.0%). Nine in ten (*n* = 336, 88.6%) had a BMI ≥ 25. A fifth (*n* = 73, 19.3%) had consumed alcohol whilst 48 (12.7%) had used tobacco products in the last 12 months. Nearly half (*n* = 173, 45.6%) scored above 75.0% on the T2D/HTN knowledge questionnaire, and 294 (78.0%) reported good to complete control over their T2D/HTN.

**TABLE 1:** Background characteristics of the study population (*n* = 379).

Characteristic	*N*	%
**Diagnosis**		
HTN	372	98.2
T2D/HTN	159	42.0
**Gender**		
Male	71	18.7
Female	308	81.3
**Age group (mean 55 ± 10.4)**		
20–49	100	26.4
50–64	208	54.9
65+	71	18.7
**Marital status**		
Never married/cohabited	72	19.0
Married/cohabiting	173	45.6
Separated/divorced/widowed	134	35.4
**Education**		
None	24	6.3
Primary	136	35.9
Secondary	142	37.5
Tertiary	77	20.3
**Occupation** †		
Employed	91	24.0
Unemployed	210	55.4
Retired	76	20.1
**Average income per household/month** ‡		
< R1500.00	59	15.6
R1500.00–R3000.00	212	55.9
> R3000.00	108	28.5
**Household size**		
1–4 members	184	48.5
5 or more members	195	51.5
**Body mass index**		
18.5–24.9 (normal)	42	11.4
25–29.9 (overweight)	74	19.5
30–34.9 (class I obesity)	84	22.2
35–39.9 (class II obesity)	79	20.8
≥ 40 (class III obesity)	99	26.1
**Current tobacco use (last 12 months)**	48	12.7
**Used alcohol in the past 12 months (yes/no)**	73	19.3
**Physically active (≥ 30 min/day) in the last week**		
1–3 days	225	84.3
4–7 days	42	5.7
**Diabetes knowledge (correct answers)**		
≤ 50%	53	19.0
51% – 75%	153	40.4
> 75%	173	45.6
**Perceived illness control**		
No/limited control	85	22.4
Good/complete control	294	77.6

HTN, hypertension; T2D, type-2 diabetes.

† , Two petty traders were excluded from analysis as their unemployment status was unclear;

‡ , South African rand (ZAR).

**FIGURE 1:**
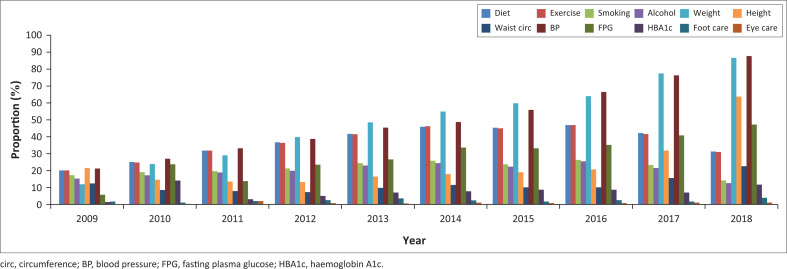
Annual risk factor monitoring (medical records audit 2009–2018).

Of the 372 available medical records, 361 (97.0%) were eligible for review. Most patients (*n* = 351, 97.2%) were prescribed antihypertensive medication in 2009, increasing yearly to 358 (99.1%) by 2018. There were 56 (35.0%) T2D patients who were prescribed chronic diabetes medication and/or insulin in 2009, increasing yearly to 150 (94.3%) by 2018. Risk factor monitoring was best for weight (86.6%) and blood pressure (87.7%). Patient’s annual weight monitoring increased from 11.9% in 2009 to 86.6% in 2018, and blood pressure monitoring increased from 21.2% in 2009 to 87.7% in 2018. Annual advice on foot care was 1.7% in 2010 compared to 3.9% in 2018 and eye screening 0.0% in 2009 compared to 2.0% in 2018) (Figure 1).

### Self-reported and medically recorded complications

Overall, complications were reported by 172 (45.4%) patients. Nearly one quarter (*n* = 84, 22.0%) of patients reported one complication, 84 (22.2%) reported two complications, and 35 (9.2%) reported three or more complications. There were 73 (20.2%) patients with a complication documented in their medical records. Medically recorded complications ranged from 11.1% (1 complication) to 4.2% (3 or more complications). (Table 2)

**TABLE 2:** Proportion of self-reported/medically recorded complications.

Number of complications	Self-reported (*N* = 379)		Medically recorded (*N* = 361)	
	*N*	%	*N*	%
0	207	54.6	288	79.7
1	84	22.2	40	11.1
2	53	13.9	18	5.0
≥ 3	35	9.2	15	4.2

### Prevalence and types of disease complications

**FIGURE 2:**
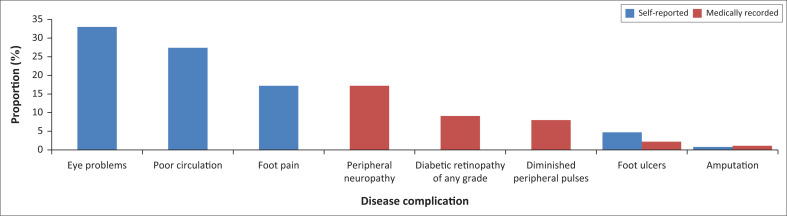
Proportion of self-reported and medically recorded complications.

The most common complications reported by participants were eye problems (*n* = 125, 33.0%) and poor circulation (*n* = 104, 27.4%), whilst the least reported complication was amputation (*n* = 3, 0.8%). From the medical records, peripheral neuropathy (*n* = 62, 16.4%) was the most prevalent complication, whilst amputation (*n* = 4, 1.1%) was least prevalent (Figure 2).

### Determinants of disease complications

Occurrence of self-reported complications was positively associated with age and female gender (Table 3). Education level, marital status, employment status and income level showed no consistent association. Tertiary education, however, was positively associated with occurrence of self-reported complications. Perceived illness control was negatively associated with occurrence of medically recorded complications. Diabetes knowledge did not show an association with self-reported complications, but it showed a weak positive association with the occurrence of medically recorded complications.

**TABLE 3:** Determinants of self-reported and medically recorded disease complications.

Variable	Self-reported complications (*n* = 379)				Medically recorded complications (*n* = 361)			
	Model 1		Model 2		Model 1		Model 2	
	Est.	s.e.	Est.	s.e.	Est.	s.e.	Est.	s.e.
Female gender	0.62**	0.30	0.52*	0.31	0.62	0.38	0.81**	0.41
Age	0.04****	0.01	0.05****	0.01	0.03**	0.01	0.02	0.02
Primary education	0.46	0.50	0.54	0.51	0.18	0.67	0.28	0.69
Secondary education	0.64	0.50	0.70	0.40	0.58	0.66	0.78	0.67
Tertiary education	0.99$	0.53	1.09*	0.53	0.76	0.69	0.81	0.70
Married or cohabiting	−0.12	0.24	−0.19	0.24	0.59**	0.29	0.52*	0.30
Unemployed	−0.43	0.28	−0.42	0.28	−0.07	0.35	−0.12	0.36
Income	−0.00008	0.0002	−0.00004	0.0002	−0.0005	0.0003	−0.0005	0.0003
Oral T2D medication	-	-	−0.60***	0.23	-	-	−0.15	0.28
Perceived illness control	-	-	−0.02	0.06	-	-	−0.35****	0.08
Diabetes knowledge†	-	-	0.13	0.10	-	-	0.23*	0.13

Note: Logit estimates of socio-demographic characteristics (Model 1); and socio-demographic characteristics and self-management variables (Model 2).

T2D - type-2 diabetes; s.e., standard error; Est., estimate.

* , *p* < 0.1;

** , *p* < 0.05;

*** , *p* < 0.01;

**** , *p* < 0.001.

† , Diabetes knowledge was only estimated for people with type-2 diabetes.

## Discussion

### Key findings

This study assessed the monitoring for disease risk factors and estimated the prevalence and factors associated with disease complications amongst T2D and HTN patients attending two CDMPs in an urban township in South Africa. A significant proportion (45%) of patients reported at least one disease complication whilst medically recorded complications were at one fifth (20%). Age, gender and perception of control over disease were associated with disease complications in this population. There was overall, poor yearly monitoring for disease risk factors in the CDMPs, but showing a gradual increase between 2009 and 2018. In this study, the best monitored risk factors were weight and blood pressure, similar to a study evaluating lifestyle care process amongst T2D patients in South Africa.^22^ Despite that, nearly 90% of the patients were overweight or obese, a known risk factor for insulin resistance and diabetes complications, thus indicating that monitoring needs to be complemented with appropriate interventions when risk factors are identified. Foot care was poorly monitored despite its importance in preventing amputation, which is one of the most feared diabetes complications contributing to delayed health seeking according to T2D patients in another township in South Africa.^23^ In spite of the high prevalence of eye problems, eye screening was also poor in these CDMPs. Similar findings were observed in the Western Cape NCD audit^12^ which attributed this to possible infrastructural and human resource challenges. We recommend that eye and foot care monitoring should be given more attention and suggest additional research to identify the barriers explaining this poor monitoring.

### Discussion of key findings

Other studies have recorded a higher prevalence of complications than ours, including a similar study performed amongst patients attending clinics in Cape Town in 1997, which recorded a prevalence of any grade of retinopathy of 55.4%, peripheral neuropathy (27.6%), and amputations (1.4%), but a lower proportion of circulation problems as indicated by absent foot pulses (8.2%).^8^ The differences could be because of their complication rates being measured in real time by study physicians rather than retrospectively reported by patients or based on medical records. Complication rates in our study are also lower than those from a Tanzanian study, which reported 49.6% (95% confidence interval [CI] 28.6–70.7) ophthalmic, and 28.8% (95% CI 8.0–65.1) neurological abnormalities,^24^ possibly reflecting health system differences, as the latter study did not report complication rates amongst patients attending CDMPs.

The positive association between female gender and having a complication remaining even after controlling for BMI was unexpected and warrants further investigation. The positive association between tertiary education and occurrence of self-reported complications was unexpected, but could be explained by more adequate reporting by this subgroup. In terms of self-management, perceived control over illness was negatively associated with medically recorded disease complications, underlying the importance of adequate self-management support. Similar findings were reported in other studies^25,26^ which assessed illness perceptions, self-care behaviours and their relationship in recently diagnosed T2D patients with and without diabetes-related complications. A possible explanation of the inconsistent association between diabetes knowledge and the number of complications could be that patients with a better knowledge are more vigilant for disease complications and more likely to report complications or seek care when a complication occurs.

### Strengths and limitations

The limitations associated with a cross-sectional survey include the lack of identification of causal relationships. Data collection was based on self-reported information and medical records. Both methods are prone to several types of measurement error (e.g. recall bias, social desirability bias, incomplete registration, etc.). Despite complications, patients may also refrain from seeking care in other facilities which may have resulted in an underestimation of the medically registered complications, especially because screening of complications was poor.

However, including information on complications from two different sources provides the option of triangulation as both sources have an added value, despite being prone to a substantial measurement error. As such, we encourage readers to look at these figures through different lenses whilst on the one hand taking into account measurement error, but including the patient and health worker’s perspective on the other hand. The association between diabetes knowledge and outcomes of interest could also have been influenced by other factors which are not measured in this study, such as adherence to treatment regimens.

### Implications or recommendations

We hypothesise that using innovative strategies towards capacitating patients to control their disease during care provision could assist with reduction of disease complications in our setting. Suggested strategies for enhancing patient self-management and control of T2D/HTN include healthcare worker capacitation to provide optimal self-management support to patients and identifying patient success stories of self-management in real life settings to encourage other patients.^27^ Examples include the use of drawings made by patients to assess their illness perception,^28^ extended engagement with patients beyond the clinical setting using interventions that include peer led support groups, psycho-behavioural support, community based lifestyle programmes for patients and their families, and cooking classes.^29^

We recommend a re-orientation of CDMPs to meet actual patient needs whereby the clubs focus on providing individualised self-management support to patients^11^ consistent with their illness experience. Risk factors identified during yearly CDMP attendance should be adequately addressed through tailored self-management interventions and subsequent audits to assess effect of the interventions.

## Conclusion

In this study, patients with comorbid T2D/HTN experienced disease complications despite attending CDMPs. Important risk factors related to feet and eyes were poorly monitored. Weight control was well monitored but poorly managed. Illness perception in terms of having control over the disease was negatively associated with prevalence of disease complications. To provide optimal care, CDMPs should make patient self-management a core aspect of their strategies, strengthen the risk factor monitoring and effectively address the identified risk factors. An evaluation component such as facility based clinical audits should be added to the CDMPs to monitor implementation of interventions.
